# Topologically Protected Edge State in Two-Dimensional Su–Schrieffer–Heeger Circuit

**DOI:** 10.34133/2019/8609875

**Published:** 2019-02-05

**Authors:** Shuo Liu, Wenlong Gao, Qian Zhang, Shaojie Ma, Lei Zhang, Changxu Liu, Yuan Jiang Xiang, Tie Jun Cui, Shuang Zhang

**Affiliations:** ^1^School of Physics and Astronomy, University of Birmingham, Birmingham B15 2TT, UK; ^2^Key Laboratory of Optoelectronic Devices and Systems of Ministry of Education and Guangdong Province, College of Optoelectronic Engineering, Shenzhen University, Shenzhen 518060, China; ^3^State Key Laboratory of Millimeter Waves, Southeast University, Nanjing 210096, China

## Abstract

Topological circuits, an exciting field just emerged over the last two years, have become a very accessible platform for realizing and exploring topological physics, with many of their physical phenomena and potential applications as yet to be discovered. In this work, we design and experimentally demonstrate a topologically nontrivial band structure and the associated topologically protected edge states in an RF circuit, which is composed of a collection of grounded capacitors connected by alternating inductors in the x and y directions, in analogy to the Su–Schrieffer–Heeger model. We take full control of the topological invariant (i.e., Zak phase) as well as the gap width of the band structure by simply tuning the circuit parameters. Excellent agreement is found between the experimental and simulation results, both showing obvious nontrivial edge state that is tightly bound to the circuit boundaries with extreme robustness against various types of defects. The demonstration of topological properties in circuits provides a convenient and flexible platform for studying topological materials and the possibility for developing flexible circuits with highly robust circuit performance.

## 1. Introduction

Topological insulators (TI), which insulate in the bulk but conduct on the surface, have been the subject of many recent studies in physics aimed at achieving topologically protected nontrivial band structures and the associated exotic phenomena, with possible implementation in diverse fields ranging from solid in electronics [1–3], ultra-cold atoms [4], microwave metamaterials [5], acoustic [6], mechanical systems [7], etc. Topologically protected edge state was originally discovered in high-purity two-dimensional electron gases (2DEGs) [8] and has recently received increasing interests in the field of photonics. In photonics, the topological insulating states are usually realized through application of external magnetic field [9] or synthetic gauge field [10]. The topologically protected edge state could be potentially used for one-way transport of light in photonics, whereas it has always been a challenge for conventional materials to transmit photons along sharp corners without scatterings. Recently, Segev's group presented a TI laser with high efficiency and extreme robustness to defects/disorder, opening an entirely new avenue to the laser design with superior performance [11, 12].

The previously demonstrated Chern and Z2 topological insulators are enabled by the presence of Berry curvatures or non-Abelian Berry curvatures. It is expected that many of the topological features found in condensed-matter physics can find their analogues in RF circuits. Many topological phenomena and properties, including the robust edge state, can be readily reproduced with electrical circuits. Importantly, topological circuits represent a highly flexible platform for investigating topological phenomena due to the convenient connections between nodes at arbitrarily long distances. This may lead to realization of 3D topological systems without introducing extra synthetic dimensions [5, 13, 14]. So far, only a few works have reported the realization of topological phenomena with LRC circuits [15–24]. In one of the earliest proofs of concept demonstrations, the topological circuits consist of a network of inductors with carefully designed connections through capacitors [15], in which RF wave experiences an effective magnetic field as they travel through each plaquette, forming a classical analogy to the Hofstadter model originally proposed in quantum mechanics [25]. They demonstrated experimentally in such an RF circuit the existence of nontrivial bandgaps containing localized edge modes. It has been recently proposed that, by stacking multiple graphene-like LRC circuit lattices into a 3D circuit, one can realize both nodal line state with drumhead-like surface bands and Weyl state with Fermi-arc-like surface bands [17, 20]. By replacing one of the capacitors in the 1D Su–Schrieffer–Heeger (SSH) circuit with two series back-to-back varactor diodes, a nonlinear circuit was demonstrated in which topological characters depend on the input intensity [19]. In addition, two recent studies reported the experimental observation of higher-order topological phases in microwave [21] and RF frequency [23, 24] circuits, both showing topologically protected corner states in analogy to the quantized bulk quadrupole moment of an electronic crystal [26].

The SSH model has attracted increasing research interests in the past decades, due to its rich physical phenomena, including topologically protected edge states, fractional charge, PT symmetry, and topological soliton excitation [27, 28]. In this work, we present the design and experimental realization of a 2D SSH quantum circuit exhibiting a Berry curvature free topologically nontrivial band structure and a topologically robust edge state.

## 2. Results

### 2.1. Model of the 2D SSH Model

**Figure 1:**
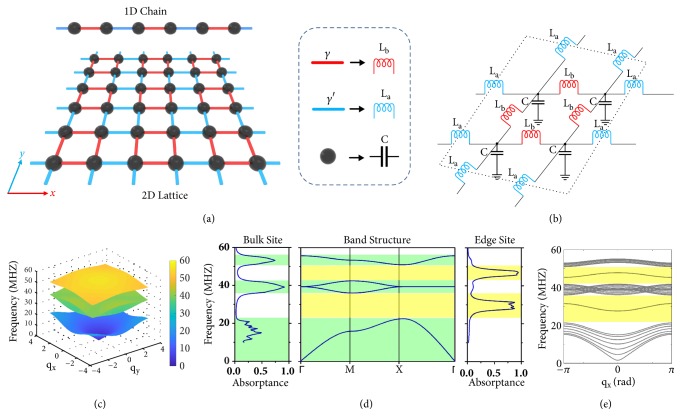
*Circuit design and band structure of the 2D SSH topological circuit. (a)* An illustration of a 2D SSH model containing 3×3 unit cells, which can be viewed as a dimerized 2D lattice with alternating hopping amplitude *γ* and* γ'*.* (b)* Unit cell of the 2D SSH circuit, obtained by replacing the potential well (black sphere) and two hopping amplitudes *γ* and* γ'*, respectively, with a capacitor and two different inductors. The inductances* L*_*a*_ and* L*_*b*_ indicate, respectively, the coupling strength between capacitors inside the unit cell (intracoupling) and between two adjacent unit cells (intercoupling).* (c)* 2D band structure theoretically calculated from the grounded circuit Laplacian of the periodic circuit model in* (b)* with the following circuit parameters:* L*_*a*_=39nH,* L*_*b*_=220nH,* C*=1000pF*. (d)* Band structure along the high symmetry lines and the numerically simulated absorption spectra on the bulk and edge sites. The frequency of the absorption peaks on both the bulk and edge sites coincides exactly with the band structure.* (e)* Band structure of the finite-sized 2D SSH circuit with 1×7.25 sites. The two curves located between the three bulk bandgaps indicate the existence of the nontrivial edge mode.

We start with a brief review of the 1D SSH model, which was originally developed to describe the 1D polyacetylene and is one of the simplest models to host topological properties [29]. The SSH model describes a chain of identical atoms with alternating strengths of bonds, as shown in the top inset of Figure 1(a). Each building block consists of two identical atoms, i.e., a dimer, with a coupling coefficient *γ* between them. The coupling coefficient between the two neighboring atoms across the unit cell boundary is* γ'*. The SSH chain exhibits a topological nontrivial phase and supports a topologically protected edge mode at the end of the chain if the intracell coupling *γ* exceeds the intercell coupling* γ'*. Next we generalize the simple configuration of the 1D SSH model into a 2D SSH circuit with alternating coupling terms *γ* and* γ'* in both* x* and* y* directions, as schematically illustrated in Figure 1(a). Note that a previous work had reported the 2D SSH model in the regime of solid state physics, which exhibits a fractional wave polarization characterized by the 2D Zak phase [30]. To realize the 2D SSH model with a realistic circuit, we replace each atom and two hopping amplitudes *γ* and* γ'*, respectively, with a capacitor and two different inductors. The unit cell of 2D SSH circuit is shown in Figure 1(b), which is composed of four identical grounded capacitors C, with every two adjacent ones connected by alternating inductors* L*_*a*_ and* L*_*b*_ in both the* x* and* y* directions.

Having described the unit cell of 2D SSH circuit, we proceed to provide the mathematical tool for analyzing its topological properties. We apply the Kirchhoff's Law to the circuit unit cell by assuming the voltages ***V*** and currents ***I*** in all the nodes and branches, as indicated in Supplementary [Supplementary-material supplementary-material-1]. To calculate the band structure, we consider an infinite 2D periodic lattice with* q*_*x*_ and* q*_*y*_ denoting the phase of Block wave vector propagating in the* x* and* y* directions, respectively. Based on [17, 20], the voltage and current vectors ***V*** and ***I*** in such a periodic circuit can be linked through a grounded circuit Laplacian ***J*** in the following form (see detailed derivation in Supplementary Materials Note 1),

(1) $$\begin{array}{c} \left(\begin{array}{c} I_{a}\\ I_{b}\\ I_{c}\\ I_{d} \end{array}\right)=J\left(\begin{array}{c} V_{a}\\ V_{b}\\ V_{c}\\ V_{d} \end{array}\right) \end{array}$$

with ***J*** being expressed as

(2) $$\begin{array}{c} J=i\omega \left(\begin{array}{cccc} C-\frac{2}{\omega^{2}L_{a}}-\frac{2}{\omega^{2}L_{b}} & \frac{e^{-iqx}}{\omega^{2}L_{a}}+\frac{1}{\omega^{2}L_{b}} & \frac{e^{-iqy}}{\omega^{2}L_{a}}+\frac{1}{\omega^{2}L_{b}} & 0\\ \frac{e^{iqx}}{\omega^{2}L_{a}}+\frac{1}{\omega^{2}L_{b}} & C-\frac{2}{\omega^{2}L_{a}}-\frac{2}{\omega^{2}L_{b}} & 0 & \frac{e^{-iqy}}{\omega^{2}L_{a}}+\frac{1}{\omega^{2}L_{b}}\\ \frac{e^{iqy}}{\omega^{2}L_{a}}+\frac{1}{\omega^{2}L_{b}} & 0 & C-\frac{2}{\omega^{2}L_{a}}-\frac{2}{\omega^{2}L_{b}} & \frac{e^{-iqx}}{\omega^{2}L_{a}}+\frac{1}{\omega^{2}L_{b}}\\ 0 & \frac{e^{iqy}}{\omega^{2}L_{a}}+\frac{1}{\omega^{2}L_{b}} & \frac{e^{iqx}}{\omega^{2}L_{a}}+\frac{1}{\omega^{2}L_{b}} & C-\frac{2}{\omega^{2}L_{a}}-\frac{2}{\omega^{2}L_{b}} \end{array}\right) \end{array}$$

**Figure 2:**
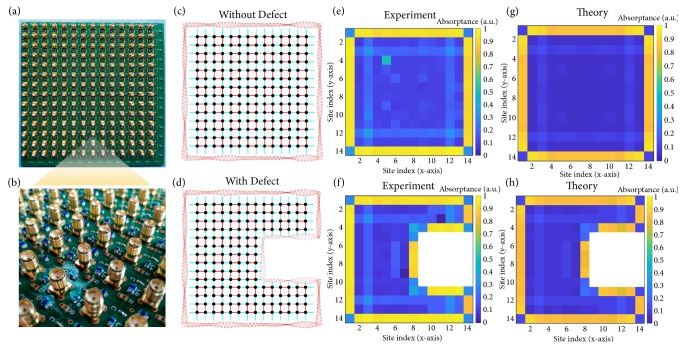
*Fabricated sample, simulation, and experimental results of the 2D SSH circuit. (a,b)* Photograph and zoomed view of the fabricated sample with 7.25×7.25 unit cells. All the boundary sites are terminated by inductors* L*_*a*_.* (c,d)* Schematic illustration of the circuits without and with defect, respectively. The blue and red lines represent inductors* L*_*a*_ and* L*_*b*_, respectively, while the black sphere represents the grounded capacitors. The red wavelike curve indicates the nontrivial edge states.* (e,f)* Experimentally measured results of the absorptance distribution at the lower bandgap (averaged between 25.2 and 28.6 MHz) for the cases without and with defect, respectively.* (g,h) *Theoretical results of the absorptance distribution at the lower bandgap (30 MHz) for the cases without and with defect, respectively.

Substituting* L*_*a*_=39nH,* L*_*b*_=220nH,* C*=1000pF into (2) and solving det[**J**(*ω*, *q*_*x*_, *q*_*y*_)] = 0, we can readily obtain the eigenvalues of the periodic circuit at different Bloch wave vectors* q*_*x*_ and* q*_*y*_, producing the 2D band structure as shown in Figure 1(c), which includes four bulk bands spanning from 0 to 55 MHz. The middle two bands are degenerate at the four corners of the first Brillouin zone (BZ) and are separated from the first and fourth bands. Figure 1(d) compares the theoretically calculated band structure and the absorption spectra on the bulk and edge sites, which are numerically calculated from a finite-sized circuit containing 7.25×7.25 sites using Agilent Design System (see method). Note that the fraction 0.25, which is the grounded capacitor, is due to the additional inductors* L*_*a*_ added to the right and bottom edges, which accounts for a quarter of unit cell (Figure 1(b)). There must be a grounded term at the edge; otherwise, the coupling component (inductors) will be unconnected. Three absorption peaks of the bulk sites (see left panel) appear exactly in the frequency range of the bulk bands in the band structure diagram (see middle panel, green regions). Two edge mode peaks can be identified from the absorption spectra of the edge site (see right panel), which resides in the two bandgaps (yellow regions). The good agreement between the band structure and the absorption spectra demonstrates the accuracy of the circuit Laplacian in analyzing the topological circuit. By varying the value of capacitors and inductors, we can tune the band gap sizes (see Supplementary [Supplementary-material supplementary-material-1]) in a wide range. While the circuit Laplacian (see (2)) for the infinitely large circuit model (Figure 1(b)) can provide all the bulk modes inside the finite-sized topological circuit, the edge modes should be calculated from the finite-sized circuit Laplacian. We present in Figure 1(e) the band structure of the finite-sized 2D SSH circuit with 1×7.25 sites supercell along the* x* direction. Two curves representing the eigenvalues of the edge mode reside between the two bandgaps marked by yellow regions, whose frequency ranges coincide exactly with the absorption peaks in the frequency spectrum (Figure 2(d)).

Although topologically protected edge states appear at the edge of topological circuit, they are closely related to the bulk states through the bulk-edge correspondence, which can predict, from the bulk circuit Laplacian, the number of topologically protected edge modes present in a finite-sized TI. To demonstrate the origin of these edge states, we calculate the topological invariant, Zak phase, from the grounded circuit Laplacian matrix* J* in (2). For the 1D SSH model, the topological phases are characterized by the winding number and determined by the intracell and intercell coupling terms *γ* and* γ'*, respectively. Specifically,* γ'*>*γ* results in a topologically nontrivial winding numbers -1 and 0 for *γ*'*<γ*, corresponding to the Zak phase of *π* (nontrivial) and 0 (trivial). Similarly, the 2D SSH circuit takes a Zak phase* π* in the regime of* L*_*a*_*<L*_*b*_, where a nontrivial edge mode appears at the four boundaries, whereas the edge mode disappears as we exchange the value of inductors (*L*_*a*_*>L*_*b*_, see Supplementary [Supplementary-material supplementary-material-1]), which corresponds to a trivial phase of zero. Note that a method was recently proposed to experimentally measure the Zak phase of an LC coupled circuit network via probing the bulk quantities [18].

We can obtain the spectrum of eigenmodes of the finite-sized circuit for different choices of* L*_*a*_ and* L*_*b*_ by calculating the eigenvalues (*ω*^*2*^) of the dynamical matrix **D** = **C**^−1/2^**WC**^−1/2^ based on the method given in [17, 20]. Here, ***C*** and ***W*** are the capacitance matrix and inverse inductivity matrix of the finite-sized circuit, respectively. Supplementary Materials Note 3 presents the detailed derivation process. In Supplementary [Supplementary-material supplementary-material-1], two separated modes (magenta color) can be clearly identified from the gaps of three bulk modes (blue color, Supplementary [Supplementary-material supplementary-material-1]), while they are absent from the trivial case when the values of* L*_*a*_ and* L*_*b*_ are exchanged (Supplementary [Supplementary-material supplementary-material-1]). These two distinct topological states cannot adiabatically transform between each other unless the bandgap closes by setting* L*_*a*_=*L*_*b*_ (Supplementary [Supplementary-material supplementary-material-1]). All the above theoretical analyses further confirm that the edge mode is not a result of a trivial surface effect, but a manifestation of the bulk nontrivial topological phase.

### 2.2. Experimental Validation of Topological Properties

It can be expected, according to the bulk-boundary correspondence, that our 2D SSH circuit supports an edge mode localized at the four edges in the nontrivial regime. To support this expectation, we design and fabricate a circuit board which incorporates 7.25×7.25 unit cells, as shown by the photographs in Figures 2(a) and 2(b), and also the schematics in Figures 2(c) and 2(d). Here, for simplicity, the capacitors and inductors are represented by the black sphere and blue/red lines, respectively. Inductors (*Q* ~37 at 40MHz) and capacitors with the same values as the numerical simulations are selected for the construction of the real sample. An SMA connector is branched out from each node to facilitate the measurement of the absorption spectra. To minimize the influence of parasitic parameters on the circuit performance and meanwhile to take into consideration the operational frequency range of the VNA (Keysight N5230C, 10MHz to 40 GHz), the value of circuit elements is deliberately chosen for the resonance to fall in the range between 10 and 60 MHz. The circuit layout is carefully designed such that the parasitic parameters (i.e., parasitic capacitances and inductances) due to the adjacent lines have negligible effect on the topological properties.

Reflectance measurement was firstly carried out to experimentally characterize the 2D SSH circuit. The absorption spectrum, which represents the amount of RF energy pumped into the circuit, can be simply obtained from 1 − *S*_11_^2^ [23]. As the reflectance coefficient* S*_*11*_ is measured with a 50 Ohm coaxial cable, it reaches zero (linear scale) when the input impedance of a certain node equals 50 Ω, leading to a maximum absorptance of unity. The expected edge mode distribution is illustrated in Figure 2(c), where the RF energy only exists around the edges of the square circuit lattice. To get a clear view of the edge mode distribution across the entire circuit lattice, we map out node by node the average absorptance at the lower (25.2-28.6 MHz, Figure 2(e)) and higher (39.5-40.6 MHz, Supplementary [Supplementary-material supplementary-material-1]) midgaps, which are consistent with the theoretical results shown in Figure 2(g) and Supplementary [Supplementary-material supplementary-material-1], respectively. From the absorptance distribution at both bandgaps, we can clearly observe bright boundaries at all the four edges. The dark corners are due to the additional grounded inductors at the four corners, which result in a blue shift of the absorptance peak in the frequency spectrum that deviates from the bandgap of the edge mode. Supplementary [Supplementary-material supplementary-material-1] presents the average absorptance distribution for the three bulk bands, where the bright pixels inside the circuit lattice represent the bulk state. Good agreement can be found between the simulated and experimentally measured absorptance (see Supplementary [Supplementary-material supplementary-material-1]), except for a frequency shift of around 8 MHz, which might be caused by the parasitic parameters of the real circuits, including the parasitic inductance of the line itself and the parasitic capacitance between neighboring lines. To clearly visualize how the amplitude of absorptance varies along the* x/y* direction on the circuit lattice, we present in supplementary [Supplementary-material supplementary-material-1] the statistical data of the absorptance for columns 1-14, where dots represent their mean value. As expected from the theoretical prediction, the edge mode decays rapidly into the bulk site.

Because such edge modes are protected by the topological nature of the SSH circuit, they are thus robust to certain types of defects and disorder. We demonstrate the robustness of the 2D SSH circuit by removing a square-shaped patch which consists of 3×3 unit cells, as illustrated in Figure 2(d). To introduce an appropriate defect to the 2D SSH circuit without affecting the Zak phase of the bulk circuit network, the circuit should be terminated in the way same as the other edges; i.e., all the edges should be terminated with an inductor* L*_*a*_ connected to the ground, such that the topology nature is still protected by the inversion symmetry of the 2D SSH circuit. As shown by Figures 2(f) and 2(h), instead of being destroyed by the defect, the edge mode persists at the edges on the newly generated boundaries of the defect regions. In addition, the spectrum of eigenmodes calculated from the dynamical matrix still shows two separated modes in the band gaps of three bulk modes (Supplementary [Supplementary-material supplementary-material-1]).

**Figure 3:**
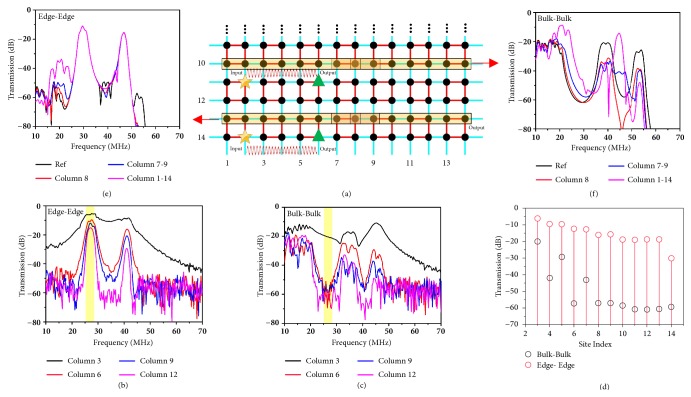
*Experimental and simulated transmission coefficients for the bulk mode and nontrivial edge mode with and without defects. (a)* Schematic illustration of the test configurations. The input port is fixed at column 2 (yellow star).* (b,c)* Transmission spectra experimentally probed as the output port is connected to columns 3, 6, 9, and 12 in the same row with the input port for the edge-to-edge case (row 14) and bulk-to-bulk case (row 11), respectively.* (d)* The average transmission (25-28 MHz, yellow region) for the edge-edge case and bulk-bulk case when port 2 is swept from columns 3-14 in the same row. The propagation loss of the edge mode is substantially lower than the bulk mode.* (e)* Numerically simulated transmission spectra for the edge mode propagating from column 2 to column 8 at the bottom row, and when different capacitors in the upper row (row 13) are connected to ground at both ends*. (f)* Numerically simulated transmission spectra for the bulk mode propagating from column 2 to column 8 at row 11 and when different capacitors in the upper row (row 10) are connected to ground at both ends.

The insulating bulk and conducting edge nature of topological insulator circuit can also be revealed by inspecting the transmission coefficients (*S*_*21*_) between two nodes on the edge and bulk. We note, in the following tests, that the RF signal is pumped into the 2D SSH circuit from the second resonator at the bottom edge with a stimulating port (port 1, indicated by the yellow star in Figure 3(b)) and, unless otherwise specified, the probing port (port 2, green triangular in Figure 3(b)) is swept across the other resonators in the same row. We select the bottom edge for the edge-to-edge test and plot in Figure 3(b) the spectra of transmission coefficients when port 2 is connected to the nodes in columns 3, 6, 9, and 12. Two obvious peaks appear at the same frequency range as in the absorption spectra ([Supplementary-material supplementary-material-1]). The transmission decreases with the increasing distance between the two ports, but all remain above the noise level with at least a 10 dB margin. However this is not the case for the bulk-to-bulk transmission as shown in Figure 3(c), which is measured at row 11 with same method. Due to the insulating nature of the topological circuits, the transmission drops more rapidly than the edge-to-edge case and almost falls to the noise level after passing column 8. To have an easy comparison of the transmission amplitude between these two cases, we present in Supplementary [Supplementary-material supplementary-material-1] their average transmissions as port 2 is swept from column 3 to column 14 in the same row. The transmission amplitude on all resonators at the edge is substantially higher than that in the bulk and most importantly with the signal in the bulk site decreasing with a rate of almost ~10 dB/node. The above test again verifies the topological insulating nature of the 2D SSH circuit.

To further demonstrate the robustness of the topologically protected edge state, we measure the transmission coefficient of the edge and bulk states under the presence of different number of small defects. As illustrated in Figure 3(a), in this test scenario, the input and output ports are fixed at columns 2 and 8 for row 11 (bulk-to-bulk) and row 14 (edge-to-edge), respectively, while we connect both ends of the capacitors to ground in row 10 and row 13, which can be viewed as the small defects. Figure 3(e) shows the transmission spectra of the edge mode measured at the bottom row when both ends of the capacitors in row 10 at column 8, columns 7-9, and columns 1-14 are grounded, as indicated by the three light yellow shaded regions. The black curve showing three transmission peaks is provided as the reference where no defect is introduced (see Figures 3(e) and 3(f)). As we ground row 8 column 8, the second and third transmission peaks drop by over 10 dB as compared to the reference curve. Further increasing the number of grounded capacitors leads to both amplitude and frequency shifts in the transmission spectra. Interestingly, for the edge mode propagating along the bottom edge, little influence is found from the transmission peaks, even in the extreme case where the capacitors in the entire row 13 are grounded at both ends, thus verifying the robustness of the nontrivial edge mode.

## 3. Discussion

In this work, we presented the design and experimental realization of a 2D SSH circuit exhibiting a nontrivial band structure and topologically protected edge state. We experimentally identified the topologically protected edge modes in a sample with 7.25×7.25 unit cells, which were located on all the edges and decayed rapidly into the bulk sites. The circuit performance is robust against component tolerance of ~5% and component Q factor of ~10 (see Supplementary Figures [Supplementary-material supplementary-material-1] and [Supplementary-material supplementary-material-1]), making the experimental realization of such 2D SSH circuit feasible at RF frequency with most commercially available capacitors and inductors. Most importantly, the nearly invariant transmission peak in the presence of different types of defects served as a clear experimental signature of topologically robust transport. Our 2D SSH topological circuit may be viewed as a location-dependent band-pass filter for RF photons travelling in a finite-sized network composed of inductive coupled capacitors. While we only presented a 2D circuit here, which is compatible with conventional circuits, we believe that such RF circuits with flexible topological connections may be particular interesting for implementing the 3D and 4D TIs which owe much richer physics than the 2D system.

The proposed topological circuit may find potential applications in flexible electronics, a technology for assembling electronic circuits on flexible substrates, such as polyimide and polyester films [31, 32]. However, one of the technical issues that hinders such flexible circuits from application is the deterioration of circuit responses when the circuit experiences different types of deformation that may lead to unexpected variation in the distributed circuit parameters (capacitance and inductance). Fortunately, topological circuits with highly robust circuit performance are superior to conventional circuits in protecting circuit functionalities from being deteriorated by physical deformations (e.g., bending, folding, twisting, compressing, and stretching) as long as they do not affect the bulk topological invariant (see Supplementary [Supplementary-material supplementary-material-1] and [Supplementary-material supplementary-material-1]).

## 4. Materials and Methods

### 4.1. Numerical Simulation

The software Agilent Design System is employed for the numerical simulation of the finite-sized 2D SSH circuit having 7.25×7.25 unit cells, which is built with the exact value as the real components selected for the fabricated sample. The simulated absorption spectra given in Figure 1(d) are on the node at row 8 column 8 as the bulk site, and the node at row 14 column 8 as the edge site.

### 4.2. Fabrication and Experiment

To minimize the loss effect to the topological properties of circuits, two inductors with 220nH±2% (Murata, LQW2BAN39NG00#) and 39nH±2% (Murata, LQW2UASR22G00#) inductances are selected, with Q-factors reaching ~38 and ~35 at 40 MHz, respectively. The self-resonance frequency of both inductors is over 500MHz, far beyond the operational frequency of our topological circuit. Chip multilayer ceramic capacitors of 1000 pF ±5% (GRM1882C1H102JA01-01A) are selected for the grounded capacitors. Keysight N5230C VNA was employed to measure the reflection and transmission coefficient, which had been calibrated using a 50 Ω calibration unit (E5052D) before measurements. The transmission coefficient between two nodes was performed by means of two-port transmission measurement using a pair of microwave cables, with one serving as the excitation and the other probing the response.

## Supplementary Materials

Supplementary Materials Figure S1: circuit schematic with the node voltages* V*_*1*_*-V*_*4*_ on the four nodes and current* I*_*1*_*-I*_*8*_ in the corresponding branches. Figure S2: bandwidth of the lower and higher bandgaps with respect to inductances* L*_*a*_* and L*_*b*_. Figure S3: numerically simulated absorption spectra of the 2D SSH circuit for the edge site when* L*_*a*_=220 nH and* L*_*b*_=39nH. Figure S4: the spectrum of eigenmodes of the circuit for different choices of* L*_*a*_ and* L*_*b*_, obtained by calculating the eigenvalues of the dynamical matrix of the finite-sized circuit. Figure S5: results of the absorptance distribution at the higher bandgap for the case without and with defect. Figure S6: distribution of average absorptance of the three bulk bands. Figure S7: experimentally measured and numerically simulated absorption spectra of the 2D SSH circuit for the bulk site and edge site. Figure S8: statistical data of the absorptance distribution in Figure 2(e) across columns 1-14, in which the dot in each column is the mean value of absorptance in each column.
