# Supplementary material for: Topologically Protected Edge State in Two-Dimensional Su–Schrieffer–Heeger Circuit
Source: Research (Wash D C). 2019 Feb 5;2019:8609875. doi: 10.34133/2019/8609875 (PMC6750084; doi:10.34133/2019/8609875)
Supplement: Supplementary Materials — Figure S1: circuit schematic with the node voltages V1-V4 on the four nodes and current I1-I8 in the corresponding branches. Figure S2: bandwidth of the lower and higher bandgaps with respect to inductances La and Lb. Figure S3: numerically simulated absorption spectra of the 2D SSH circuit for the edge site when La=220 nH and Lb=39nH. Figure S4: the spectrum of eigenmodes of the circuit for different choices of La and Lb, obtained by calculating the eigenvalues of the dynamical matrix of the finite-sized circuit. Figure S5: results of the absorptance distribution at the higher bandgap for the case without and with defect. Figure S6: distribution of average absorptance of the three bulk bands. Figure S7: experimentally measured and numerically simulated absorption spectra of the 2D SSH circuit for the bulk site and edge site. Figure S8: statistical data of the absorptance distribution in Figure 2(e) across columns 1-14, in which the dot in each column is the mean value of absorptance in each column. [file 8609875.f1.docx]

**Supplementary Materials for**

**Topologically protected edge state in two dimensional SSH circuit**

Shuo Liu^1,3†^, Wenlong Gao^1,†^, Qian Zhang^2,†^, Shaojie Ma^1,3^, Lei Zhang^2^, Changxu Liu^1^, Yuan Jiang Xiang^3^, Tie Jun Cui^2,*^, Shuang Zhang^1,*^

^1^School of Physics and Astronomy, University of Birmingham, Birmingham B15 2TT, United Kingdom

^2^State Key Laboratory of Millimeter Waves, Southeast University, Nanjing 210096, China

^3^Key Laboratory of Optoelectronic Devices and Systems of Ministry of Education and Guangdong Province, College of Optoelectronic Engineering, Shenzhen University, Shenzhen 518060, China

Corresponding authors: Y. J. Xiang: [yjxiang@szu.edu.cn](mailto:yjxiang@szu.edu.cn); T. J. Cui: [Tjcui@seu.edu.cn](mailto:Tjcui@seu.edu.cn); S. Zhang: [S.Zhang@bham.ac.uk](mailto:S.Zhang@bham.ac.uk)

**This PDF file includes:**

Supplementary Text Note.1 to Note 3

Supplementary Figures S1 to S8

**Note 1. Derivation of the Laplacian matrix of the infinite 2D SSH circuit**

Let us assume in Figure S1 the voltages *V_1_-V_4_* on the four ground capacitors and direction of currents *I_1_-I_8_* in the circuit branches. Applying the [Kirchhoff's](http://www.baidu.com/link?url=wpq-Wn7YO-rAzlWZvd5qMzfcrnagI-mv4eamhaoBaZqFf4Qlp6WM4ZMNcASTUzIBI1hGqkkrrPae1ZqidoCmYqtlB_qlvMqWf-qt3Ac3OYqIopFXlrfKQjYua2kVGdag) current Law to the four nodes, we have,

$I_{1}+I_{3}=I_{5}+I_{7}+\frac{\partial}{\partial t}V_{a}C$ (S1)

$I_{5}+I_{4}=I_{1}e^{iq_{x}}+I_{8}+\frac{\partial}{\partial t}V_{b}C$ (S2)

$I_{2}+I_{7}=I_{6}+I_{3}e^{iq_{y}}+\frac{\partial}{\partial t}V_{c}C$ (S3)

$I_{6}+I_{8}=I_{2}e^{iq_{x}}+I_{4}e^{iq_{y}}+\frac{\partial}{\partial t}V_{d}C$ (S4)


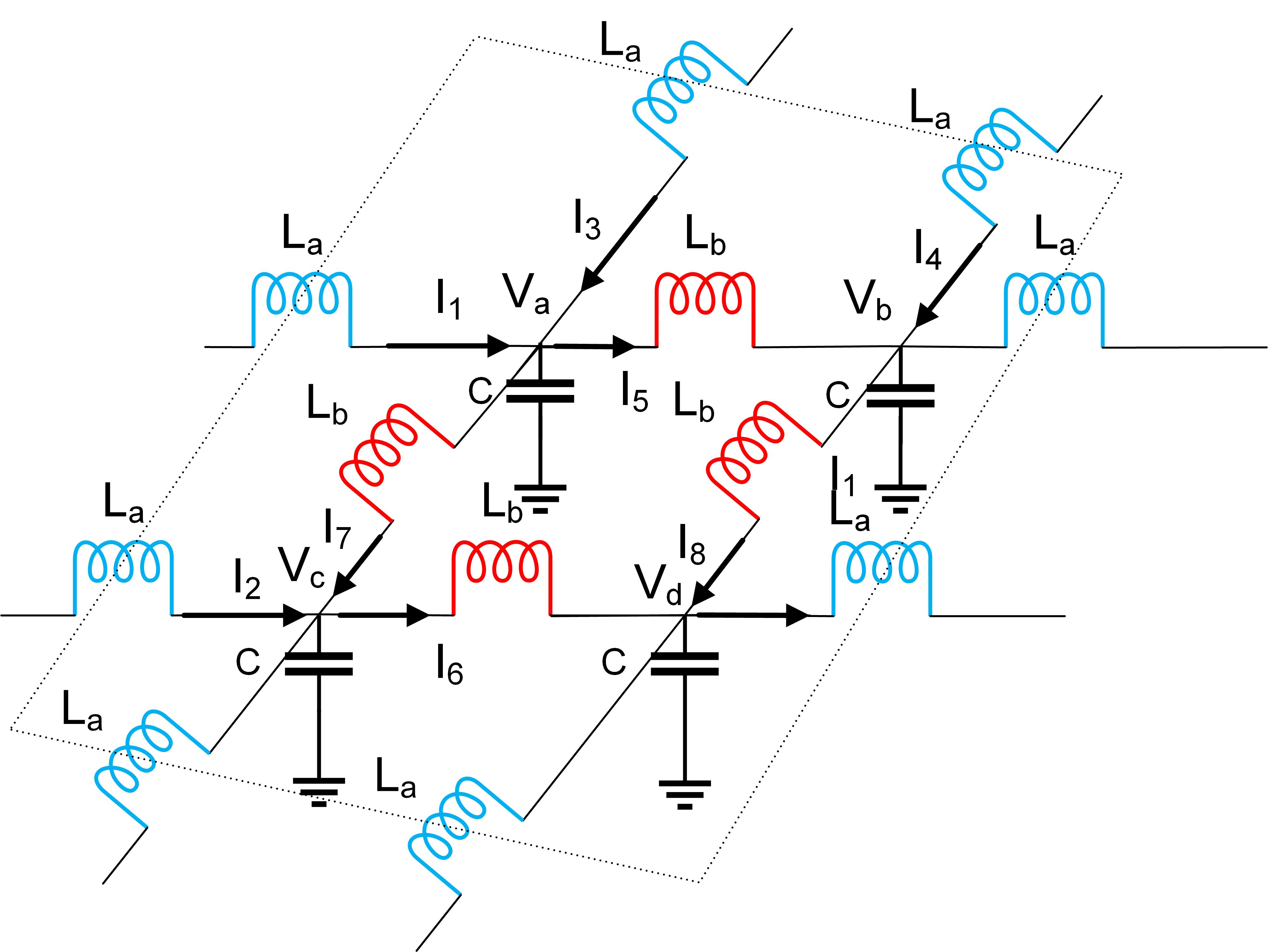


**Figure S1** Circuit schematic with the node voltages *V_1_-V_4_* on the four nodes, and current *I_1_-I_8_* in the corresponding branches.

According to the current direction given in Figure S1, we can write the current that flows out of each node as,

$I_{a}=\frac{-\left(V_{b}e^{-iq_{x}}-V_{a}\right)}{i\omega L_{a}}+\frac{\left(V_{a}-V_{b}\right)}{i\omega L_{b}}-\frac{\left(V_{c}e^{-iq_{y}}-V_{a}\right)}{i\omega L_{a}}+\frac{\left(V_{a}-V_{c}\right)}{i\omega L_{b}}+V_{a}i\omega C$ (S5)

$I_{b}=\frac{-\left(V_{a}-V_{b}\right)}{i\omega L_{b}}+\frac{\left(V_{b}-V_{a}e^{-iq_{x}}\right)}{i\omega L_{a}}-\frac{\left(V_{d}e^{-iq_{y}}-V_{b}\right)}{i\omega L_{a}}+\frac{\left(V_{b}-V_{d}\right)}{i\omega L_{b}}+V_{b}i\omega C$ (S6)

$I_{c}=\frac{-\left(V_{d}e^{-iq_{x}}_{a}-V_{c}\right)}{i\omega L_{a}}+\frac{\left(V_{c}-V_{d}\right)}{i\omega L_{b}}-\frac{\left(V_{a}-V_{c}\right)}{i\omega L_{b}}+\frac{\left(V_{c}-V_{a}e^{iq_{y}}\right)}{i\omega L_{a}}+V_{c}i\omega C$ (S7)

$I_{d}=\frac{-\left(V_{c}-V_{d}\right)}{i\omega L_{b}}+\frac{\left(V_{d}-V_{c}e^{iq_{x}}\right)}{i\omega L_{a}}-\frac{\left(V_{b}-V_{d}\right)}{i\omega L_{b}}+\frac{\left(V_{d}-V_{b}e^{iq_{y}}\right)}{i\omega L_{a}}+V_{d}i\omega C$ (S8)

Writing Eqs. (S5)-(S8) in the following form,

$\left(\begin{matrix} I_{a} \\ I_{b} \\ I_{c} \\ I_{d} \end{matrix}\right)=J\left(\begin{matrix} V_{a} \\ V_{b} \\ V_{c} \\ V_{d} \end{matrix}\right)$ (S9)

where ***J*** is the grounded Laplacian matrix,

$J=i\omega\left(\begin{matrix} \text{C}-\frac{2}{\omega^{2}L_{a}}-\frac{2}{\omega^{2}L_{b}} & \frac{e^{-iqx}}{\omega^{2}L_{a}}+\frac{1}{\omega^{2}L_{b}} & \frac{e^{-iqy}}{\omega^{2}L_{a}}+\frac{1}{\omega^{2}L_{b}} & 0 \\ \frac{e^{iqx}}{\omega^{2}L_{a}}+\frac{1}{\omega^{2}L_{b}} & \text{C}-\frac{2}{\omega^{2}L_{a}}-\frac{2}{\omega^{2}L_{b}} & 0 & \frac{e^{-iqy}}{\omega^{2}L_{a}}+\frac{1}{\omega^{2}L_{b}} \\ \frac{e^{iqy}}{\omega^{2}L_{a}}+\frac{1}{\omega^{2}L_{b}} & 0 & \text{C}-\frac{2}{\omega^{2}L_{a}}-\frac{2}{\omega^{2}L_{b}} & \frac{e^{-iqx}}{\omega^{2}L_{a}}+\frac{1}{\omega^{2}L_{b}} \\ 0 & \frac{e^{iqy}}{\omega^{2}L_{a}}+\frac{1}{\omega^{2}L_{b}} & \frac{e^{iqx}}{\omega^{2}L_{a}}+\frac{1}{\omega^{2}L_{b}} & \text{C}-\frac{2}{\omega^{2}L_{a}}-\frac{2}{\omega^{2}L_{b}} \end{matrix}\right)$ (S10)

We can obtain calculate the Zak phase of the 2D SSH circuit by firstly calculate the inner product of every two neighboring eigenstates of the J matrix, and then calculate the angle of their product,

$\gamma=-\arg\left(\left\langle\Psi_{1}\left|\Psi_{2}\right.\right\rangle\left\langle\Psi_{2}\left|\Psi_{3}\right.\right\rangle\ldots\left\langle\Psi_{N}\left|\Psi_{1}\right.\right\rangle\right)$ (S11)

A recent work demonstrated that it is equivalent to calculate the topological invariant from the circuit Hamiltonian, which can be built from the capacitance, inductance and conductance matrix. [1]

**Note. 2 Calculating of the spectrum of eigenmodes for the finite-sized circuit**

According to Refs. 2 and 3, the capacitance matrix ***C*** and inverse inductivity matrix ***W*** for a finite-sized circuit should be firstly created, which describe the connections between each adjacent node and the grounded condition of themselves. The off-diagonal components are simply the capacitance/inverse inductance between each adjacent nodes, while the diagonal components are the total node capacitance/inverse inductance as given below,

$Y_{mm}=-Y_{m0}-\sum_{n=1,2,...} Y_{mn}$ (S12)

where *Y_m0_* is the capacitance/inverse inductance between node *m* and the ground.

Based on the [Kirchhoff's](http://www.baidu.com/link?url=wpq-Wn7YO-rAzlWZvd5qMzfcrnagI-mv4eamhaoBaZqFf4Qlp6WM4ZMNcASTUzIBI1hGqkkrrPae1ZqidoCmYqtlB_qlvMqWf-qt3Ac3OYqIopFXlrfKQjYua2kVGdag) current Law, the total current flowing into (or out of) a node should be zero, hence, we have

 (S13)

Where *Φ(t)* is the voltage on each note. Assuming *Φ(t)* varies in the form of *e^jωt^*, Eq.(S13) can be simplified as,

 (S14)

If we make a gauge transformation $D\text{=}C^{-1/2}WC^{-1/2}$ and, Eq. (S14) will have the following form,

 (S15)

Now the spectrum *ω^2^* of eigenmodes can be readily obtained by calculating the eigenvalues of the dynamical matrix ***D.***

**Note. 3 Experimental and simulation results**

To present how the value of inductors affects the band structure of the 2D SSH circuit, we provide in Figure S2 the bandwidth of the lower and higher mid-gaps (yellow region in Figure 1d) as a function of inductances *L_a_ and L_b_*. As the inductances *L_a_ and L_b_* are swept from 2nH to 200nH, we observe that both the bandwidth of the lower and higher bandgaps increases with the increasing difference between *L_a_ and L_b_*, as indicated by the two bright regions close to the upper and left edges. Careful comparison of Figure S2a and b shows that the bandwidth of the lower mid-gap is larger than that of the higher band.


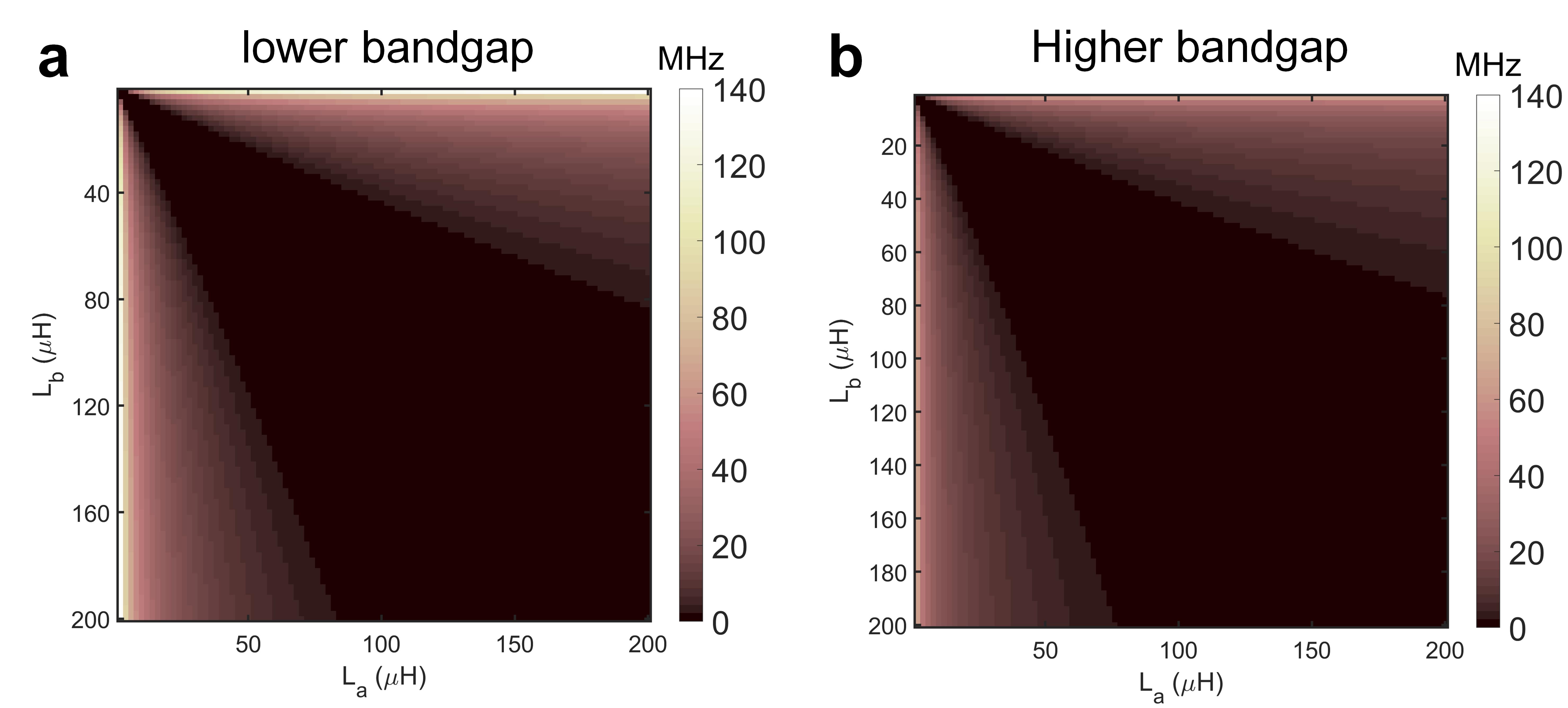


**Figure S2** Bandwidth of the lower and higher bandgaps with respect to inductances *L_a_ and L_b_*. **(a)** Lower bandgap. **(b)** Higher bandgap.


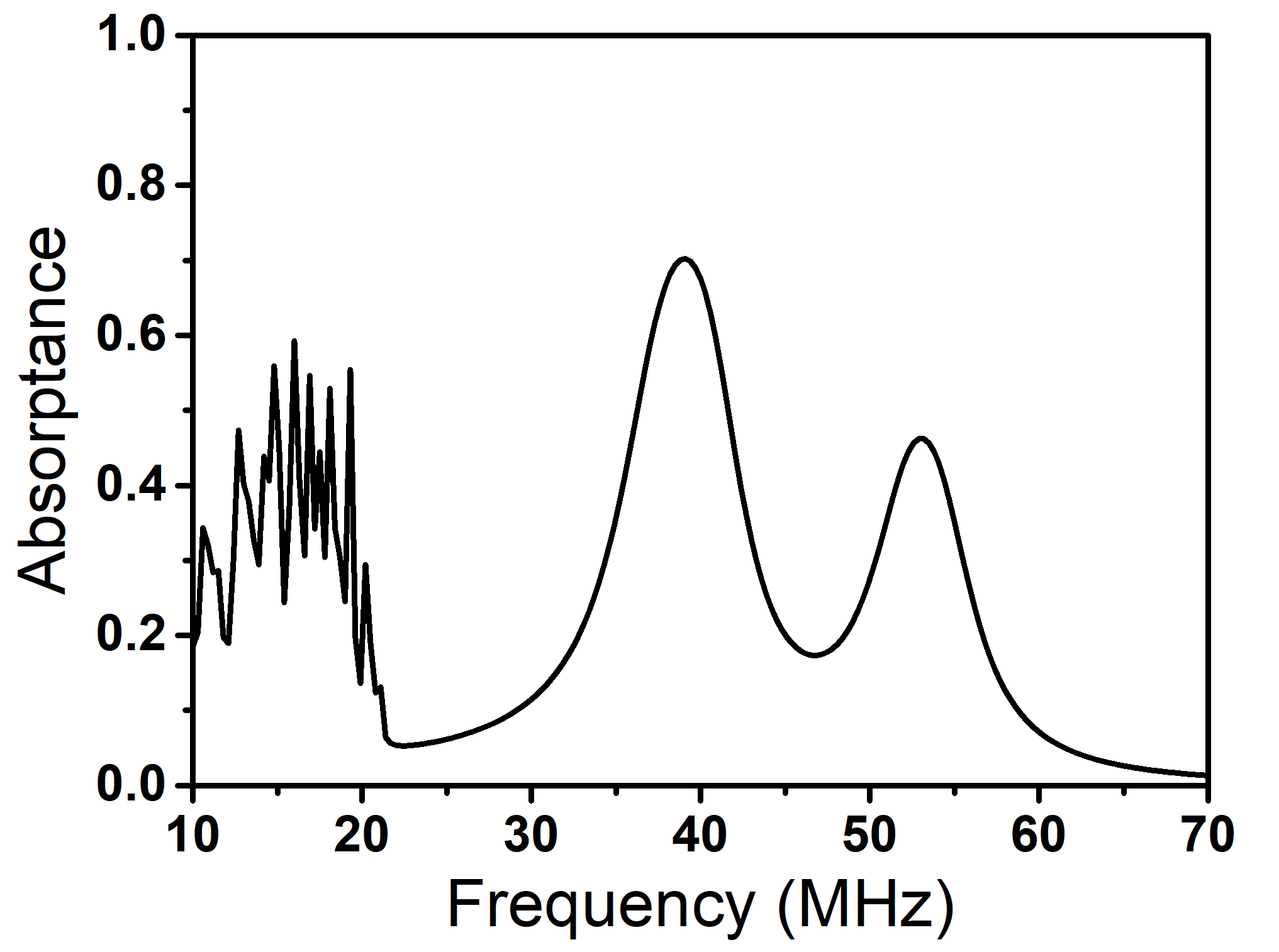


**Figure S3** Numerically simulated absorption spectra of the 2D SSH circuit for the edge site when *L_a_*=220 nH and *L_b_*=39nH.


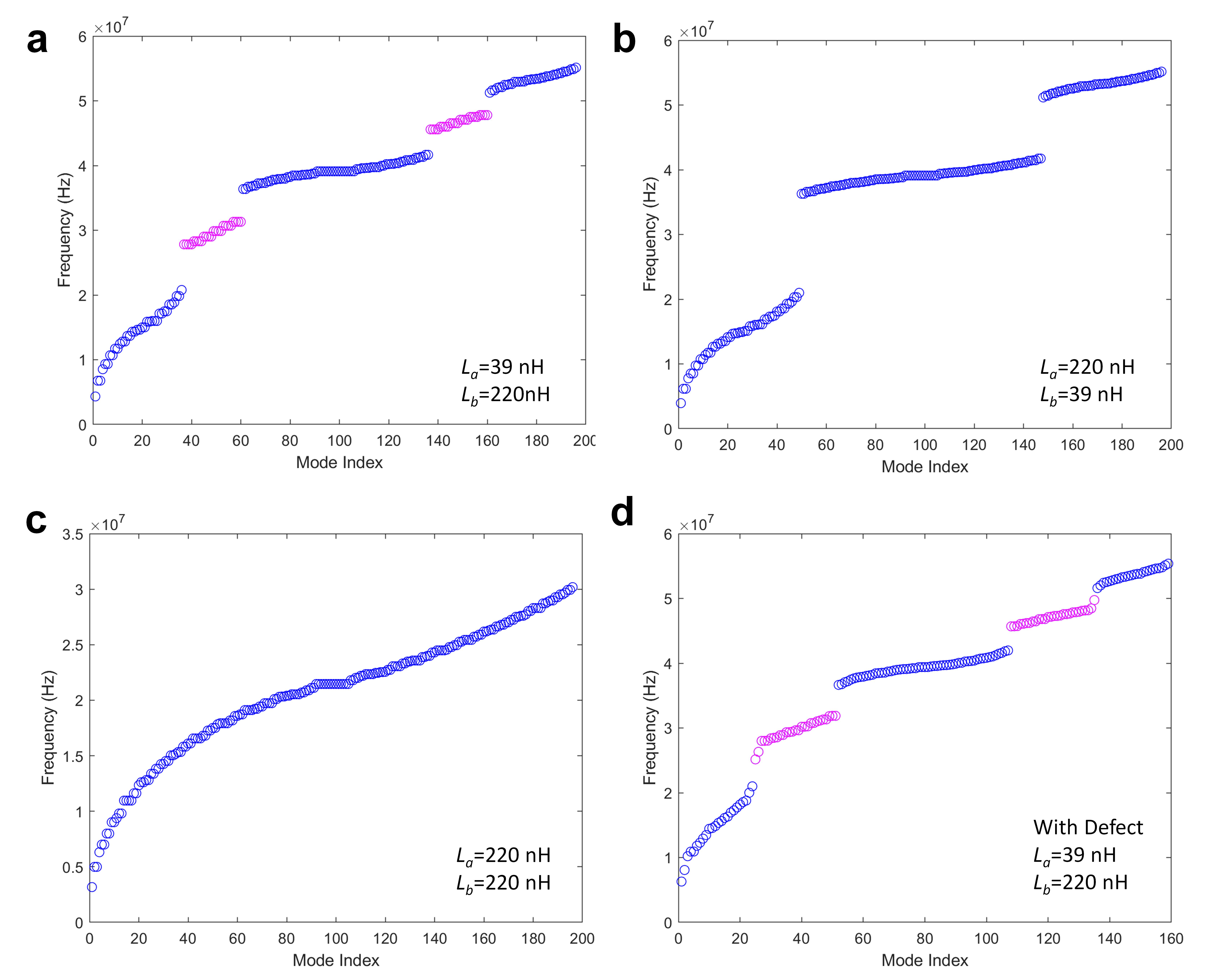


**Figure S4** The spectrum of eigenmodes of the circuit for different choices of *L_a_* and *L_b_*, obtained by calculating the eigenvalues of the dynamical matrix of the finite-sized circuit. **(a)** *L_a_*=39 nH, *L_b_*=220nH. **(b)** *L_a_*=220 nH, *L_b_*=39nH. **(c)** *L_a_*=220 nH, *L_b_*=220nH. **(d)** *L_a_*=39 nH, *L_b_*=220nH, with defect.


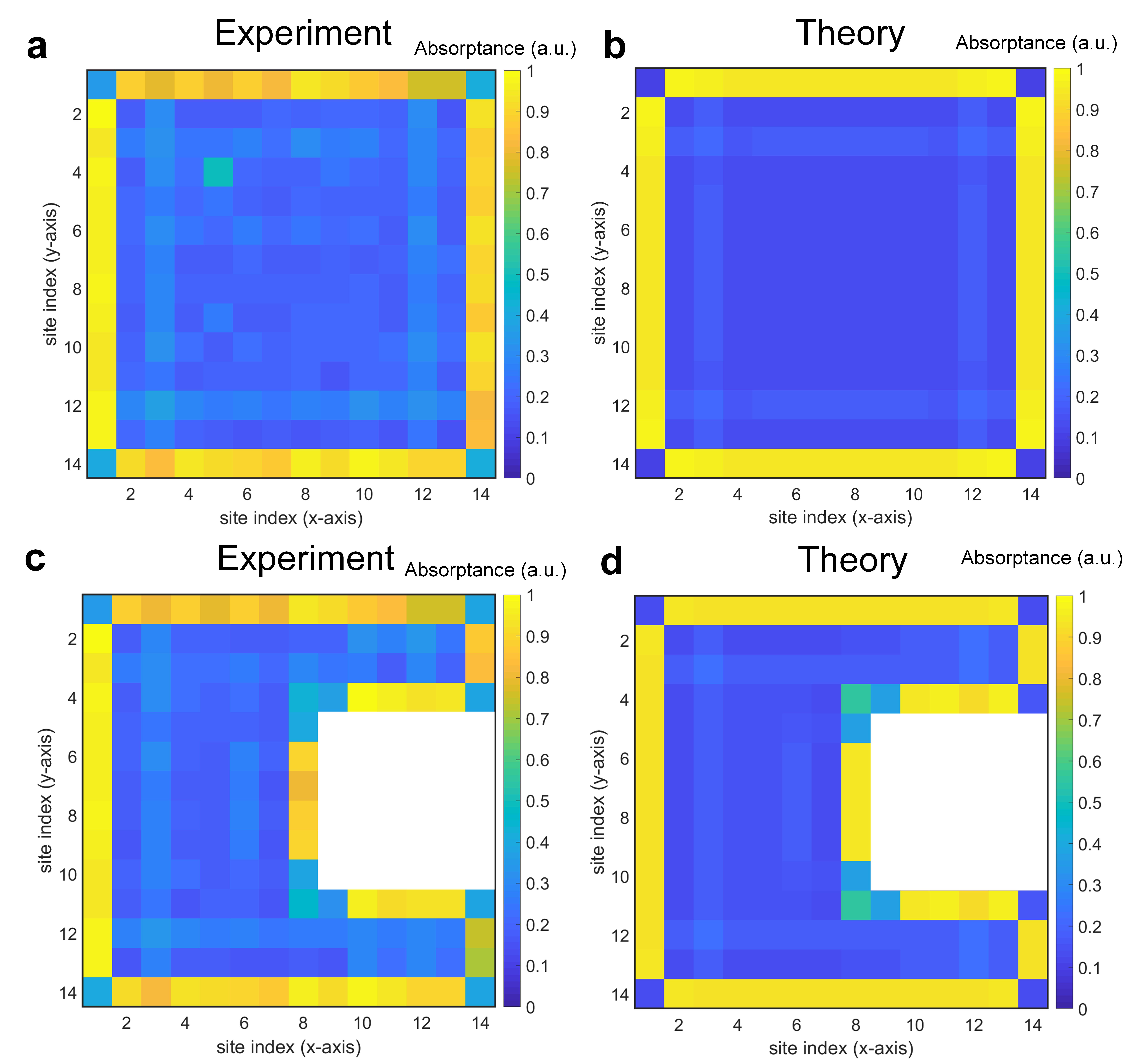


**Figure S5** Results of the absorptance distribution at the higher bandgap for the case without and with defect. **(a,c)** Experimentally measured results of the absorptance distribution at the higher bandgap (averaged between 39.5-40.6 MHz) for the case without and with defect, respectively. **(b,d)** Theoretical results of the absorptance distribution at the lower bandgap (46.5 MHz) for the cases without and with defect, respectively.


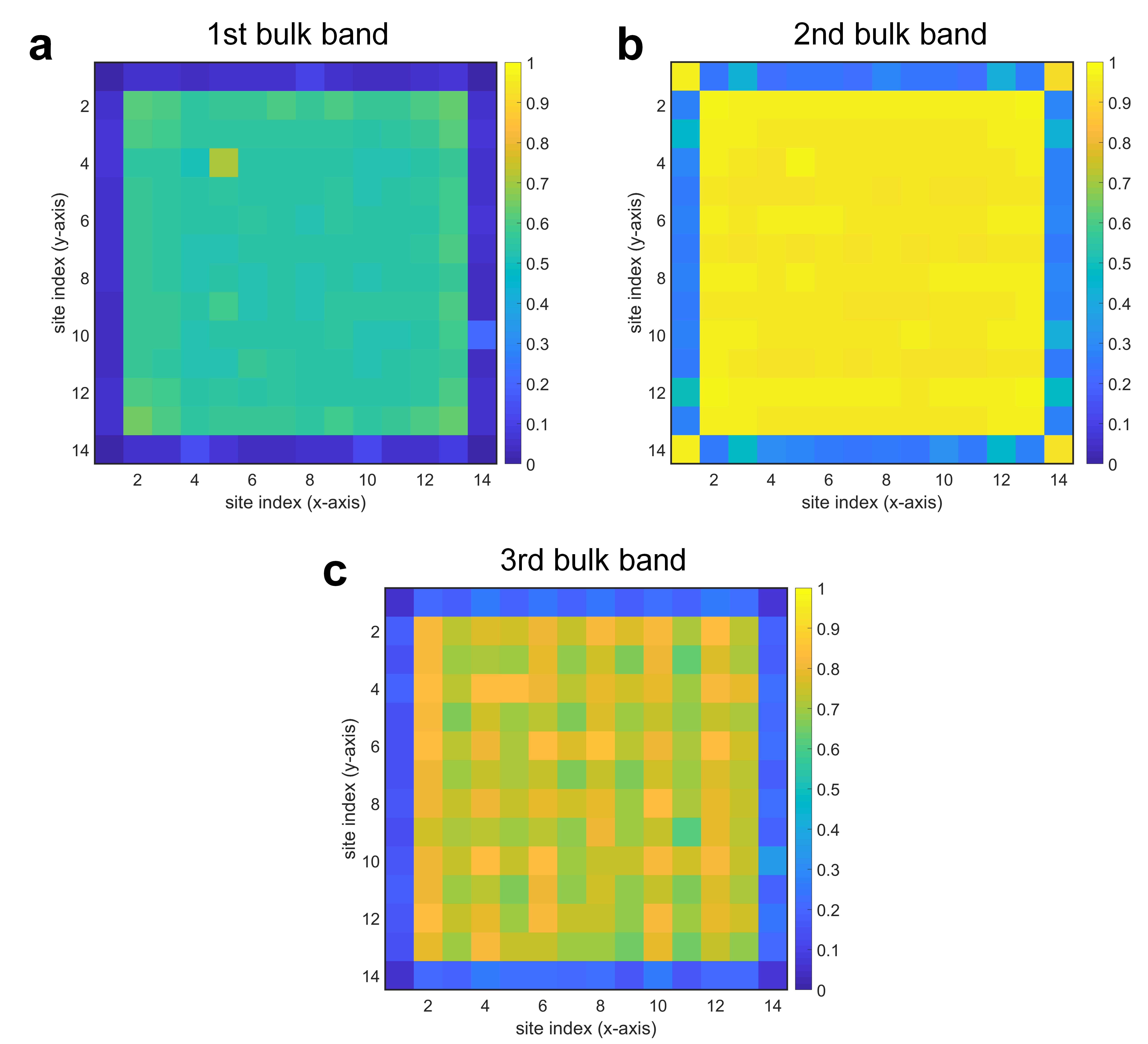


**Figure S6** Distribution of average absorptance of the three bulk bands. **(a)** First bulk band, 12-18.5 MHz; **(b)** Second bulk band, 34-36 MHz; **(c)** Third bulk band, 45-47 MHz.


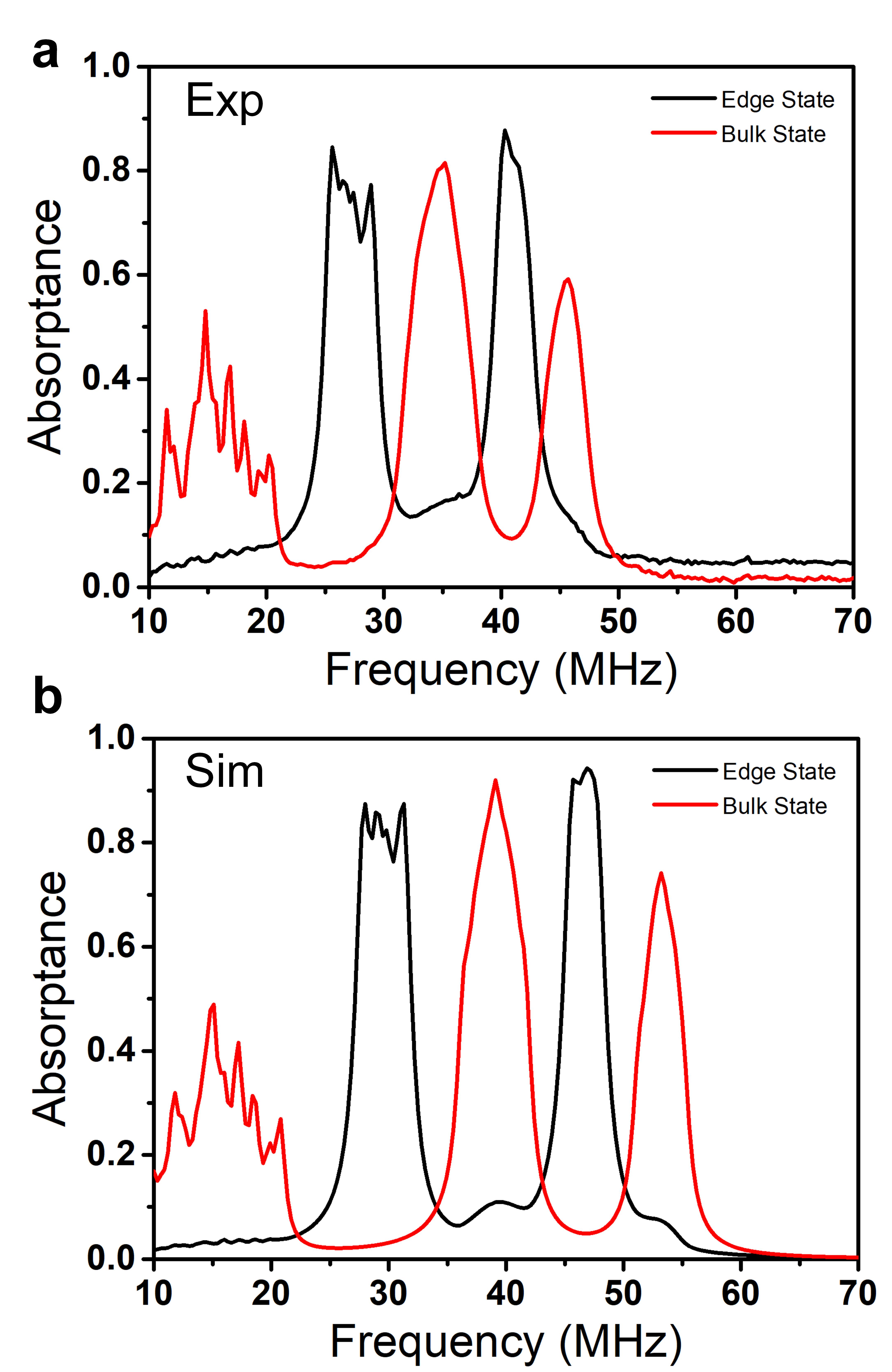


**Figure S7** Experimentally measured and numerically simulated absorption spectra of the 2D SSH circuit for the bulk site and edge site. **(a)** Experiment. **(b)** Simulation.


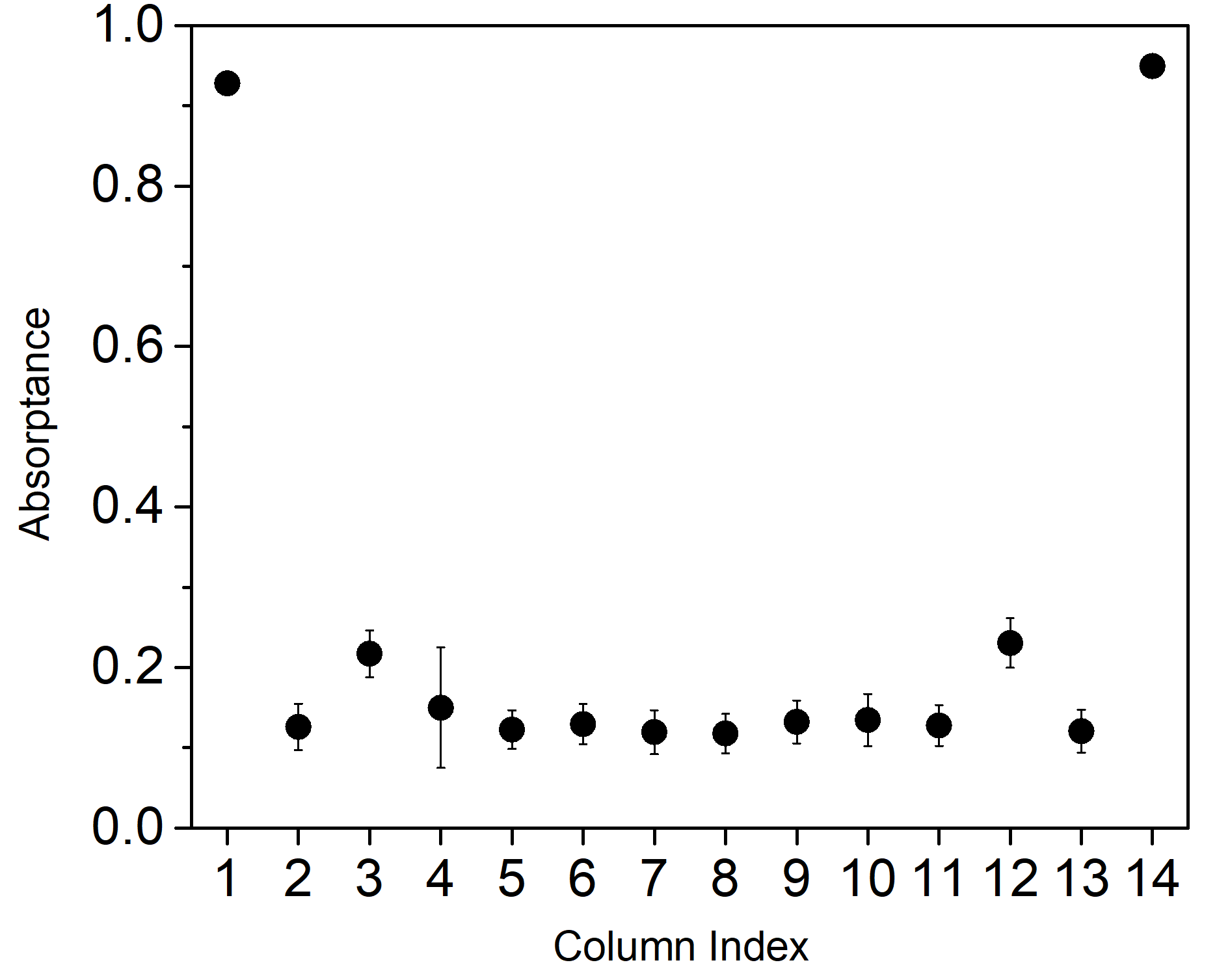


**Figure S8** Statistical data of the absorptance distribution in Figure 2e across column 1-14, in which the dot in each column is the mean value of absorptance in each column.


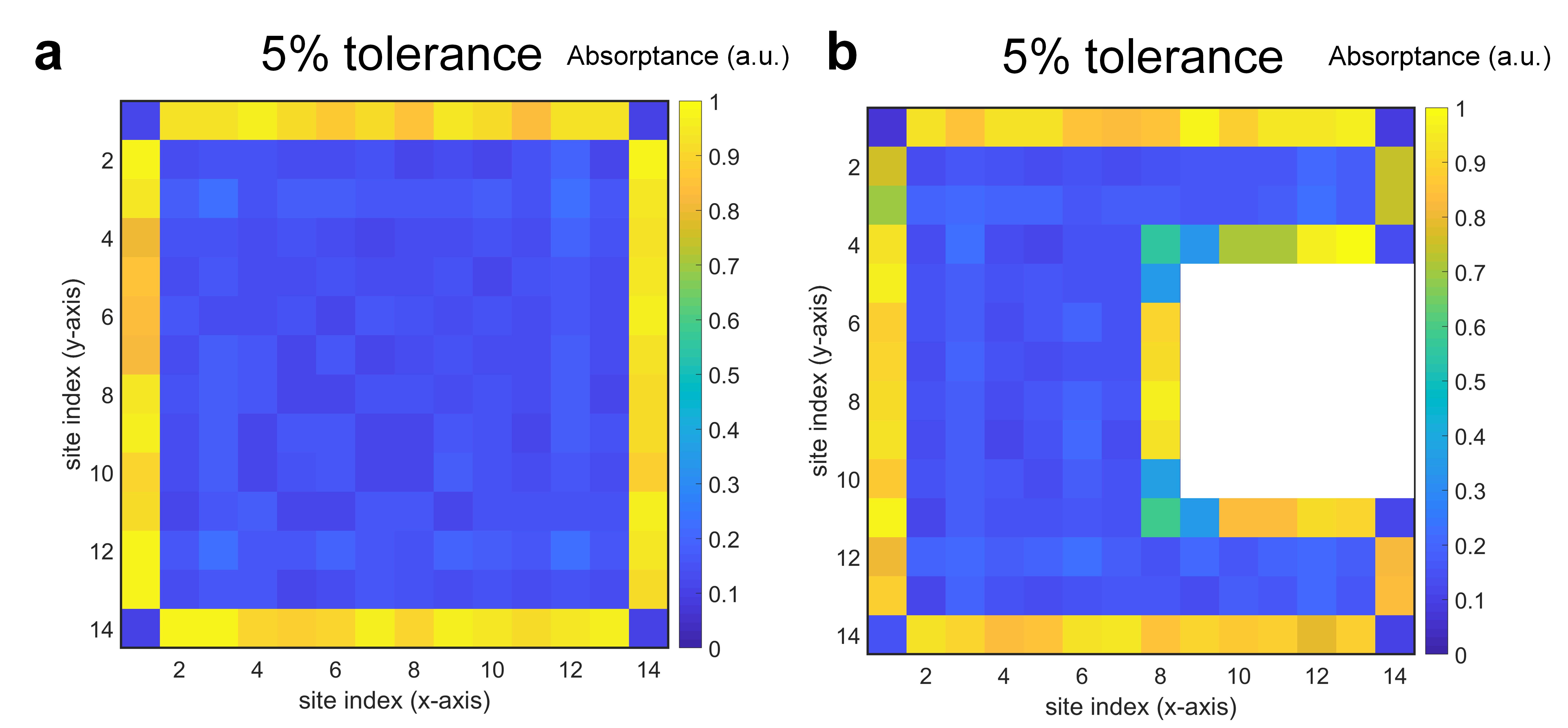


**Figure S9** Distribution of average absorptance at the higher bandgap (39.5-40.6 MHz) for the cases with 5% tolerance of capacitance and inductance. (a) Without defect. (b) With defect.


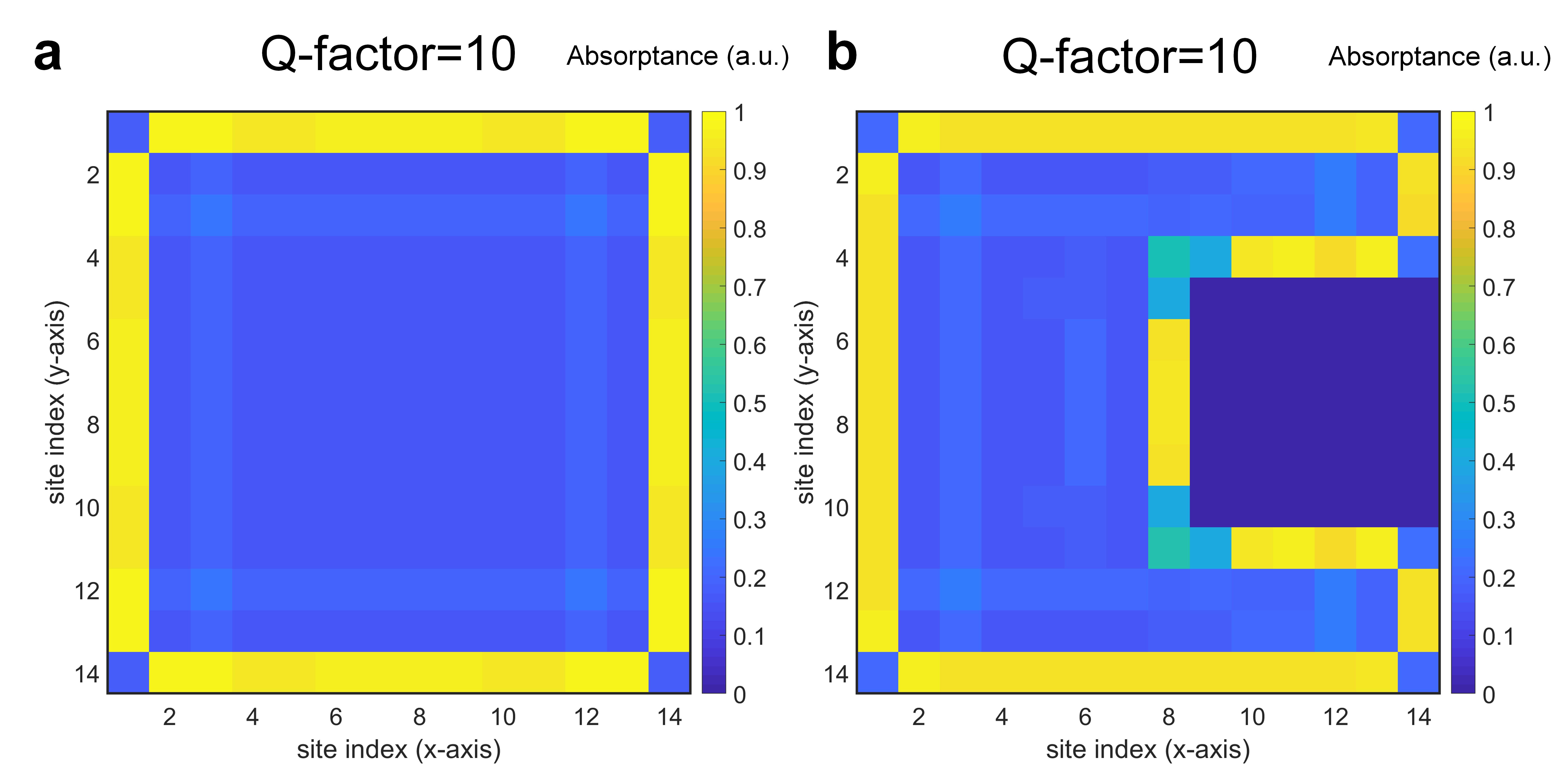


**Figure S10** Distribution of average absorptance at the higher bandgap (39.5-40.6 MHz) for the cases with Q factor of ~10 for capacitors and inductors. (a) Without defect. (b) With defect.

**Reference：**

[1] T. Hofmann, T. Helbig, C. H. Lee, et al., Chiral voltage propagation in a self-calibrated topolectrical Chern circuit. arXiv:1809.08687v1, 2018.

### [2] K. F. Luo, R. Yu, H. M. Weng, Topological nodal states in circuit lattice, *Research*, vol. 2018, <https://doi.org/10.1155/2018/6793752>, 2018.

[3] C. H. Lee, S. Imhof, C. Berger, F. Bayer, Topolectrical circuits, *Nature Communication Physics*, vol. 1, pp. 38, DOI: 10.1038/s42005-018-0035-2, 2018.
